# Einführung digitaler Lehre im Fach Psychiatrie als Reaktion auf COVID-19: eine vergleichende Evaluation zur Präsenzlehre

**DOI:** 10.1007/s00115-021-01081-5

**Published:** 2021-03-03

**Authors:** Matthias Besse, Jens Wiltfang, Michael Belz, Jörg Signerski-Krieger

**Affiliations:** 1 Klinik für Psychiatrie und Psychotherapie, Universitätsmedizin Göttingen, v. Siebold-Str. 5, 37075 Göttingen, Deutschland; 2 Deutsches Zentrum für Neurodegenerative Erkrankungen e. V. (DZNE), Göttingen, Deutschland; 3 iBiMED, Medical Sciences Department, Universität von Aveiro, Aveiro, Portugal

**Keywords:** Lernerfolg, Universitäre Lehre, Pandemie, Podcasts, Medizinstudium, Learning success, University teaching, Pandemic, Podcasts, Medical studies

## Abstract

**Hintergrund:**

Aufgrund der Corona-Pandemie musste die klassische universitäre Präsenzlehre kurzfristig auf ein digitales Format für das Sommersemester 2020 (SoSe20) umgestellt werden. Am Beispiel der psychiatrischen Klinik der Universitätsmedizin Göttingen sollten der Lernzuwachs und die inhaltliche Bewertung vergleichend zwischen beiden Lehrformen evaluiert werden, um die Qualität der Umstellung beurteilen zu können.

**Material und Methoden:**

Insgesamt 350 Studierende beurteilten die von ihnen besuchte Präsenzlehre (WiSe18/19 bis WiSe19/20) bzw. die neu etablierte digitale Lehre (SoSe20) im Rahmen einer standardisierten Lehrevaluation. Sie machten hierbei Angaben zu ihrem persönlichen Lernzuwachs in 7 psychiatrischen Kernbereichen und bewerteten die jeweilige Lehrform inhaltlich auf 8 Dimensionen. Zudem gaben sie ihren durchschnittlichen Zeitaufwand an.

**Ergebnisse:**

Die Studierenden schätzten ihren Lernzuwachs in der digitalen Lehre auf allen Dimensionen mindestens gleichwertig zur Präsenzlehre bzw. signifikant besser in den Teilbereichen „Psychotherapie“ sowie „Schizophrenie“ ein. Trotz eines signifikant erhöhten zeitlichen Aufwandes wurde die digitale Lehre auf allen Dimensionen inhaltlich gleichwertig oder besser („Selbstständiges Aufarbeiten von Lernzielen“, „Format der Vorlesung“) eingeschätzt. Bei der Vorbereitung auf die berufliche Praxis zeigten sich die Studierenden bez. der digitalen Lehre skeptisch.

**Diskussion:**

Eine kurzfristige pandemiebedingte Umstellung der Präsenzlehre hin zur digitalen Lehre führte in der hier vorliegenden vergleichenden Evaluation nicht zu einem Qualitätsverlust. Mit Blick auf die spätere praktische ärztliche Tätigkeit sollten in zukünftigen Kurrikula neben der klassischen Präsenzlehre auch digitale Lehrangebote ergänzend verankert werden.

## Abstract (en)

**Background:**

In response to the coronavirus pandemic, most universities implemented digital teaching at short notice for the summer semester 2020 (SS20), whereas they simultaneously shut down classroom teaching. In the psychiatric clinic of the University Medical Center Göttingen, students’ ratings concerning the learning effect and their substantive assessment for both forms of teaching were comparatively evaluated to determine the quality of this process.

**Material and methods:**

Overall, 350 students who had visited classroom teaching (winter semester, WS18/19 to WS19/20) vs. digital teaching (SS20) assessed their form of teaching post hoc, within a standardized survey. They rated the individual learning effect in seven psychiatric subjects and did a substantive assessment on eight dimensions. In addition, they rated their expenditure of time.

**Results:**

For digital teaching, the individual learning effect was rated as either being equivalent or superior (subjects: psychotherapy, schizophrenia). Despite a significantly heightened expenditure of time, digital teaching was substantively assessed as being equivalent to classroom teaching or superior (dimensions: independent processing of learning goals, overall format of lecture). Concerning their anticipated preparation for the professional practice, students rated digital teaching as being inferior to classroom teaching.

**Conclusion:**

A pandemic-driven conversion from classroom to digital teaching did not result in a loss of quality on the dimensions measured in this comparative evaluation. With a view to professional practice, digital teaching should complement classroom teaching and be part of future curricula.

## Hintergrund und Fragestellung

Die Corona-Pandemie hat seit dem Frühjahr 2020 zu großen Herausforderungen und Belastungen bei der täglichen Patientenversorgung geführt [1]. Zahlreiche ärztliche Fort- und Weiterbildungsangebote, so auch erstmals der Kongress der Deutschen Gesellschaft für Psychiatrie und Psychotherapie, Psychosomatik und Nervenheilkunde (DGPPN) im November 2020, mussten aufgrund der Kontaktbeschränkungen in ausschließlich digitaler Form durchgeführt werden. Auch die universitäre Lehre musste innerhalb kürzester Zeit an die neuen, der Pandemie geschuldeten Rahmenbedingungen angepasst werden. Am 02.04.2020 einigten sich die Bundesländer darauf, die Präsenzpflicht im Lehrbetrieb für das Sommersemester 2020 aufzuheben. Gleichzeitig wurden die Universitäten aufgefordert, die Voraussetzungen für die Durchführung der Lehrveranstaltungen in digitaler Form zu schaffen und das Semester auf diese Art abzuhalten [5].

Digitale Lehrangebote wie Podcasts (Audio- und/oder Videoaufzeichnungen von Vorlesungen oder anderen Veranstaltungen), Apps oder interaktive Lernspiele (sog. „serious games“) sind prinzipiell verfügbar. Allerdings scheinen diese Techniken noch nicht in der Breite zum Einsatz zu kommen und die Verwendung stark abhängig von der jeweiligen Fachrichtung und Universität zu sein [6]. Dabei gibt es gerade für den Einsatz von Podcasts – entweder als Ersatz zur klassischen Vorlesung oder als Ergänzung hierzu – Hinweise auf ein hohes Level an Akzeptanz und Nutzung seitens der Studierenden [2]. Auch scheint der studentische Lernerfolg bei der Verwendung von Podcasts mit dem klassischen Vorlesungsbesuch vergleichbar zu sein [10].

Die Präsenzlehre im Fach Psychiatrie und Psychotherapie findet an der Universitätsmedizin Göttingen (UMG) im 5. klinischen Semester statt und besteht aus den Formaten Vorlesung, Seminar und dem Unterricht am Krankenbett (UAK; Tab. 1; [4]). Während in den Vorlesungen die theoretischen Inhalte des Fachs vermittelt werden, sollen die drei Seminartermine („Psychopathologischer Befund“, „Psychiatrische Notfälle“, „Stigmatisierung“) der Wissensvertiefung in Kleingruppen dienen. Im UAK treten die Studierenden schließlich in direkten Kontakt mit den Patienten und sollen das erworbene Wissen selbstständig in der Praxis anwenden. Dieser Aufbau entspricht didaktisch der 4‑stufigen Lernpyramide nach Miller [8]. Bis zum Sommersemester 2020 wurden an unserer Klinik keine Podcasts oder vergleichbare Formate, außer einigen Patienten-Beispielvideos in Vorlesungen und Seminaren, eingesetzt.

**Table 1:** 

Präsenzlehre (WiSe18/19 bis WiSe19/20)	Angepasste digitale Lehre (SoSe 20)
*Klassische Vorlesung im Hörsaal*	*Podcasts der jeweiligen Vorlesung*
– Patientenvorstellung im Rahmen der Vorlesung	Beispielvideo mit Schauspielpatient
*Seminare in Kleingruppen*	*Inverted-classroom-Konzept*
– Psychopathologischer Befund	Kurzer Podcast mit Theorie
– Psychiatrische Notfälle	Bearbeiten der gestellten Aufgaben
– Stigmatisierung	Rückmeldung durch Dozierende
*Unterricht am Krankenbett*	*In Präsenz am Ende des Semesters durchgeführt*

Da empirische Daten zur Güte einer sehr kurzfristigen Anpassung hin zur digitalen Lehre, wie durch die Corona-Pandemie notwendig geworden, praktisch nicht existieren, war die Skepsis bei vielen Lehrenden groß: Neben einem drohenden Qualitätsverlust durch die fehlende direkte Interaktion zwischen Lehrenden und Studierenden wurde eine geringere Nutzung der digitalen Angebote erwartet. Auch ein reduzierter Lernerfolg durch die privaten und/oder beruflichen Belastungen im Rahmen der Corona-Pandemie wurde befürchtet – zudem eine schlechtere Vorbereitung auf die psychiatrischen Inhalte des kommenden Staatsexamens und die zukünftige ärztliche Tätigkeit.

Die vorliegende Studie soll am Beispiel der psychiatrischen Klinik der Universitätsmedizin Göttingen hierzu einen empirischen Beitrag leisten und die Frage beantworten, ob eine solche kurzfristige Umstellung hin zur digitalen Lehre ohne Qualitätsverlust möglich ist: Es wurde, basierend auf den Angaben von 350 Studierenden, eine vergleichende Evaluation zwischen Präsenzlehre der letzten drei Semester und der im Sommersemester 2020 etablierten digitalen Lehre, bezogen auf (a) *Lernzuwachs* und (b) *inhaltliche Bewertung*, durchgeführt^1^.

## Methodik

### Stichprobe und Studiendesign

Es wurden insgesamt 350 Studierende im 5. klinischen Semester in die vorliegende Evaluation mittels Fragebogen einbezogen. Die Studierenden konnten den beiden Subgruppen (1) *Präsenzlehre* vs. (2) *digitale Lehre* zugeordnet werden:

Für *Präsenzlehre* wurden die drei vergangenen Semester mit insgesamt 175 Studierenden einbezogen (Teilnahme Lehrevaluation: WiSe 18/19 *n* = 67 von 147, SoSe19 *n* = 58 von 142, WiSe 19/20 *n* = 50 von 142), für die digitale Lehre das aktuelle Semester (Teilnahme Lehrevaluation SoSe20, *n* = 175 von 179). Die Befragung wurde einmalig als Post-hoc-Erhebung durchgeführt und erfolgte sowohl für die Präsenzlehre in Papierform als auch für die digitale Lehre als Onlinefragebogen mittels EvaSys (Electric Paper Evaluationssysteme). Da die Erhebung im Rahmen der standardisierten Lehrevaluation an der UMG durchgeführt wurde, durften für die vorliegende Stichprobe keine demographischen Daten (Geschlecht, Alter etc.) sowie Prüfungsergebnisse erhoben werden, um die Anonymität der Studierenden zu wahren. Auch eine Auswertung der vorhandenen Freitextkommentare wurde für die aktuelle Studie aus diesem Grund nicht durchgeführt.

### Umsetzung der digitalen Lehre

Sämtliche Vorlesungen wurden in Form von Podcasts aufgezeichnet. Hierzu wurde das Programm Camtasia Studio (TechSmith) eingesetzt: Es ermöglicht die Videoaufzeichnung der PowerPoint-Präsentationen der Vorlesungen, inklusive eingesprochener Audiospur durch die Dozierenden. Die durchschnittlich 30–45 min langen Podcasts wurden schließlich im MP4-Format den Studierenden über die universitätseigene Onlineplattform Stud.IP (GPL) zur Verfügung gestellt. Die Videos waren über die gesamte Dauer des Semesters verfügbar.

Zur Umsetzung der drei Seminartermine wurde auf das Konzept des „inverted classroom“ in leicht modifizierter Form zurückgegriffen: Der eigentlichen Präsenzveranstaltung (z. B. Vorlesung oder Seminar) wird hierbei eine Selbstlernphase der Studierenden vorgeschaltet. Hierdurch soll das aktive Lernen gefördert werden [13]. Es wurde daher für jeden der drei Seminartermine ein maximal 15 min langer Podcast aufgezeichnet und hierüber ein kurzer theoretischer Input vermittelt. Am Ende der Podcasts bearbeiteten die Studierenden eine oder mehrere Aufgaben zum jeweiligen Thema selbstständig:

Für das Seminar „Psychopathologischer Befund“ (PPB) wurden Lehrvideos eingesetzt, um verschiedene psychopathologische Auffälligkeiten (u. a. Aufmerksamkeitsdefizit‑/Hyperaktivitätsstörung [ADHS], Depression, Schizophrenie) mit der Hilfe von Schauspielern darzustellen. Anschließend sahen sich die Studierenden eins der zur Auswahl stehenden Anamnesevideos [14] an und verfassten einen Kurzarztbrief mit psychopathologischem Befund. Im Seminar „Psychiatrische Notfälle“ wurden mittels des Podcasts die allgemeinen Grundsätze zum Umgang mit Notfällen vermittelt und anhand von drei Beispielen aus der klinischen Praxis verdeutlicht. Die Studierenden sollten weitere zwei schriftlich vorliegende Beispiele psychiatrischer Notfälle selbstständig bearbeiten und ein Vorgehen zur Bewältigung des jeweiligen Notfalls vorschlagen. Zum Thema „Stigmatisierung“ erfolgte ebenfalls ein kurzer Input per Podcast, um die Studierenden für das Thema zu sensibilisieren. Außerdem wurde ein für den Seminartermin neu gedrehtes Video gezeigt, in welchem eine Arzt-Patienten-Interaktion simuliert wurde. Aufgabe der Studierenden war es, die im Gespräch auftretenden Merkmale für Stigmatisierung zu identifizieren und zu bewerten.

Alle verschriftlichen Aufgaben wurden per E‑Mail an die jeweilige Seminarleitung geschickt und korrigiert. Abschließend erfolgte ein individuelles schriftliches Feedback an die Studierenden. Das in Stud.IP integrierte Forum ermöglichte den Studierenden anschließend inhaltliche Rückfragen sowie das Abgeben von Feedback.

### Fragebogen zur Lehrevaluation

Der verwendete Fragebogen enthielt insgesamt 16 Items zur vergleichenden Evaluation, die in der vorliegenden Studie als primäre Endpunkte für die Bereiche *Lernzuwachs* und *inhaltliche Bewertung* definiert sind. Von diesen waren 15 Items als Statements formuliert, die mittels 6‑stufiger Likert-Skala mit zwei Ankern (1 = „trifft voll zu“, 6 = „trifft überhaupt nicht zu“) beantwortet werden konnten. Zudem wurden die durchschnittlichen Arbeitsstunden pro Woche erfasst.

Die Bewertung des *Lernzuwachses* basierte hierbei auf einem etablierten Fragebogen [9]. Die Messung erfolgte mittels 7 paarweiser Items (ausgewählte Lernziele für den Fachbereich Psychiatrie), mit denen die Studierenden ihren Zuwachs einschätzten. Hierzu gaben sie sowohl die *rückblickende* Selbsteinschätzung zu Modulbeginn als auch die *aktuelle* Selbsteinschätzung für 7 auf die Psychiatrie bezogene Bereiche an: (a) psychopathologischer Befund, (b) Medikation bei ADHS, (c) psychotherapeutische Verfahren, (d) Demenzen, (e) Antidepressiva, (f) Schizophrenie, (g) Angststörungen^2^.

Die *inhaltliche Bewertung* wurde mit 8 Items erfasst. Die Studierenden konnten Statements zur inhaltlichen Güte des Unterrichts mittels Likert-Skala bewerten: (a) Umsetzung der Interdisziplinarität (bezogen auf die interdisziplinäre Lehre der am Modul beteiligten Fachrichtungen), (b) Förderung des selbstständigen Aufarbeitens von Lernzielen, (c) auf berufliche Zukunft bezogener Lernzuwachs, (d) Zufriedenheit mit Modulstruktur, (e) praktische Umsetzung der Lehre, (f) Wunsch nach Fortführung des Moduls in dieser Form, (g) Lernerfolg durch Vorlesungen, (h) Lernerfolg durch Seminare^3^.

Zudem wurden 7 Items zur deskriptiven Analyse exklusiv für die digitale Lehre in den Fragebogen integriert. Die Studierenden konnten hier ihre subjektiven Einschätzungen der digitalen Lehrform im Vergleich zur Präsenzlehre mit der oben beschriebenen Likert-Skala abgeben: (a) Lernzuwachs, (b) Präferenz (für digitale Lehre), (c) erhöhter Zeitaufwand, (d) Vorbereitung auf das Examen, (e) Vorbereitung auf den Beruf^4^. Außerdem schätzten die Studierenden ein, ob durch die Corona-Pandemie (f) eine Verringerung des Lernerfolges oder (g) eine generell erhöhte Belastung bestand.

### Statistische Auswertung

Die Analyse der Daten erfolgte mit der Statistiksoftware SPSS®, Version 26. Es wurden zur deskriptiven Darstellung Mittelwerte (M), Standardabweichungen (SD) und Pearson-Korrelationen berechnet (r). Für die Einschätzung des *Lernzuwachses* wurden 7 allgemeine lineare Modelle (GLM) für messwiederholte Daten erstellt, mit Integration der Selbsteinschätzung zum Leistungsstand („rückblickende Selbsteinschätzung“ vs. „aktuelle Selbsteinschätzung“) als 2‑stufiger Innersubjektfaktor. Weiterhin wurde die Lehrform („digitale Lehre“ vs. „Präsenzlehre“) als 2‑stufiger Zwischensubjektfaktor in die jeweiligen GLM integriert. Zudem wurde die Teststatistik für die Interaktion beider Faktoren für jede GLM berechnet, um mögliche Unterschiede im Lernzuwachs zwischen beiden Lehrformen zu identifizieren (Lehrform × Selbsteinschätzung). Um Unterschiede bei der einmalig erhobenen *inhaltlichen Bewertung* zwischen den beiden Lehrformen zu analysieren, wurden multiple t‑Tests für unabhängige Stichproben berechnet („digitale Lehre“ vs. „Präsenzlehre“). Die Festlegung des Signifikanzniveaus wurde aufgrund der α‑Fehler-Inflation nach der Bonferroni-Methode für die Anzahl von insgesamt 16 statistischen Tests adjustiert (7 Items: *Lernzuwachs*, 8 Items: *Inhaltliche Bewertung*, 1 Item: Zeitaufwand; kritischer *p*-Wert < 0,003). Zudem erfolgte, ausgehend von diesem Signifikanzniveau, die Adjustierung für alle berichteten Paarvergleiche innerhalb jedes einzelnen GLM. In einzelnen Fällen machten die Studierenden unvollständige Angaben im Fragebogen oder ließen Items aus – siehe die Freiheitsgrade der einzelnen Modelle bzw. statistischen Tests für die jeweils gültigen eingeschlossenen Fälle.

## Ergebnisse

### Nutzung und subjektive Einschätzung der digitalen Lehre

Von 182 in Stud.IP angemeldeten Accounts für die psychiatrische Lehre wurden von 103 (57 %) die Vorlesungspodcasts, von 137 (75 %) die Seminarpodcasts abgerufen. Da über einen Account mehrere Studierende die Videos gemeinsam ansehen konnten, liegen die tatsächlichen Nutzungszahlen potenziell höher.

**Figure 1:**
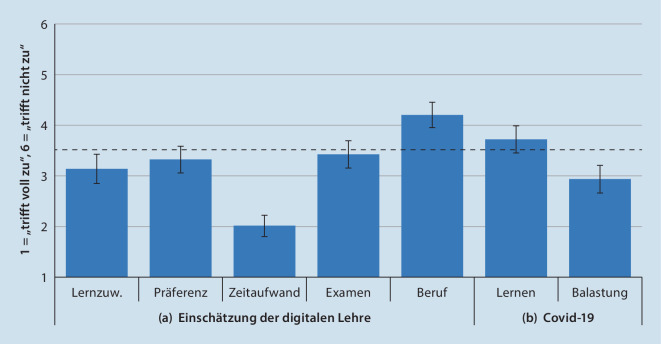

Die deskriptive Auswertung ist in Abb. 1 dargestellt. Die Studierenden in der digitalen Lehre schätzten diese tendenziell als gleichwertig im Lernzuwachs ein (M = 3,14, SD = 1,72) bzw. zeigten eine gleichwertige Präferenz zu dieser Lehrform (M = 3,32, SD = 1,66). Der Zeitaufwand wurde als erhöht eingeschätzt (M = 2,01, SD = 1,34), die Vorbereitung auf das Examen als gleichwertig (M = 3,42, SD = 1,55). Die Vorbereitung auf den ärztlichen Beruf schätzten die Studierenden tendenziell bei der digitalen Lehre als schlechter ein (M = 4,20, SD = 1,52). Bezogen auf die Corona-Pandemie gaben die Studierenden an, subjektiv nicht in ihrem Lernerfolg beeinträchtigt worden zu sein (M = 3,72, SD = 1,64), jedoch eine erhöhte Belastung (M = 2,93, SD = 1,77) wahrzunehmen.

### Vergleichende Evaluation: Lernzuwachs

Für die Gesamtstichprobe konnten, unabhängig von der Lehrform, numerische Lernzuwächse für alle 7 abgefragten Bereiche festgestellt werden (Tab. 2, *M* = −1,66 bis −2,61). Dieser Zuwachs war in hohem bis sehr hohem Maße signifikant, von „(b) Medikation ADHS“ (GLM: F[1, 327] = 521,74, *p* < 0,001, partielles η^2^ = 0,62) bis zur höchsten Ausprägung im Bereich (a) „Psychopathologischer Befund“ (GLM: F[1, 327] = 1285,60, *p* < 0,001, partielles η^2^ = 0,80). Eine steigende Anzahl an wöchentlichen Arbeitsstunden korrelierte mit erhöhten Lernzuwächsen in allen Bereichen, verfehlte hier jedoch teilweise die Signifikanz (r = −0,04 bis −0,16). Zudem korrelierte digitale Lehre signifikant mit erhöhten wöchentlichen Arbeitsstunden (r = 0,40; s. Tab. 2 für eine Übersicht).

**Table 2:** 

Variable	1	2	3	4	5	6	7	8	M ± SD/Freq
1. Digital									j: 175, n : 175
2. Arbeitsstunden	0,396^**^								32,11 ± 12,97
3. ∆ PPB	0,022	−0,043							−2,36 ± 1,41
4. ∆ Med. ADHS	−0,157^**^	−0,047	0,389^**^						−1,66 ± 1,34
5. ∆ Psychotherapie	−0,184^**^	−0,082	0,404^**^	0,472^**^					−2,00 ± 1,37
6. ∆ Demenz	−0,139^*^	−0,039	0,372^**^	0,458^**^	0,429^**^				−2,38 ± 1,31
7. ∆ Antidepressiva	−0,152^**^	−0,093	0,333^**^	0,431^**^	0,430^**^	0,557^**^			−1,98 ± 1,24
8. ∆ Schizophrenie	−0,169^**^	−0,164^**^	0,405^**^	0,514^**^	0,536^**^	0,602^**^	0,570^**^		−2,61 ± 1,39
9. ∆ Angststörungen	−0,155^**^	−0,071	0,431^**^	0,483^**^	0,580^**^	0,573^**^	0,520^**^	0,704^**^	−2,41 ± 1,27

*M* Mittelwert, *SD* Standardabweichung, *Freq* Frequenz, *PPB* psychopathologischer Befund, *Med. ADHS* Medikation bei Aufmerksamkeitsdefizit‑/Hyperaktivitätsstörung

Digital: 0 = Präsenzlehre, 1 = digitale Lehre; Arbeitsstunden für das Modul in Zeitstunden; ∆ Items 3 bis 9: Delta der Lernzuwachseinschätzung Prä- zu Postmodul (∆ = Post-Wert – Prä-Wert) für 7 ausgewählte Lernziele im Fachbereich Psychiatrie, z. B. „Ich kann die verschiedenen Formen der Angststörungen unterscheiden“: aktuelle Selbsteinschätzung vs. rückblickende Selbsteinschätzung zu Modulbeginn (1 = „trifft voll zu“ bis 6 = „trifft überhaupt nicht zu“); *n* = 350; df = 264–348

*p < 0,05; **p < 0,01

**Figure 2:**
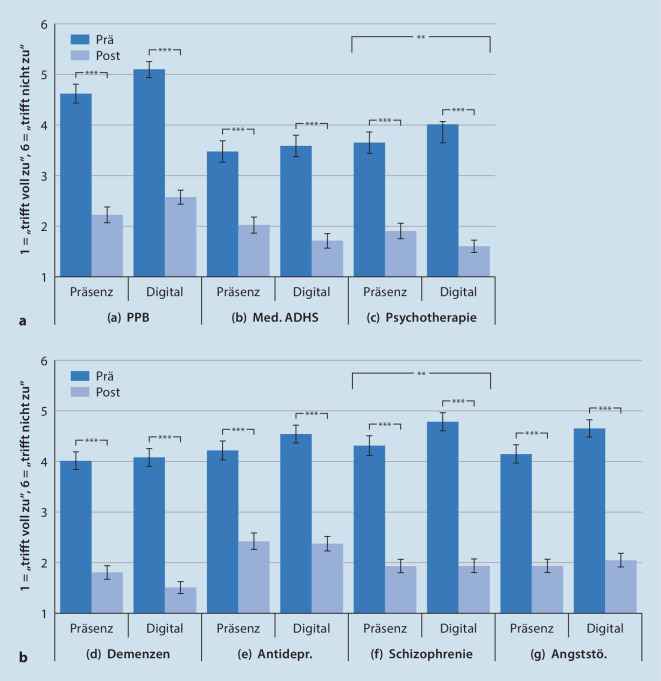

Einzeln betrachtet zeigten sich sowohl für die Präsenzlehre als auch für die digitale Lehre durchweg signifikante Lernzuwächse in allen Bereichen. Auf der verwendeten Skala (1 = „trifft voll zu“, 6 = „trifft überhaupt nicht zu“) schätzten sich die Studierenden vor Modulbeginn als wenig kompetent bez. der angestrebten Lernziele ein (Präsenzlehre: M = 4,62–3,48; digitale Lehre: M = 5,10–3,86). Diese Werte verbesserten sich zum Zeitpunkt nach der Modulteilnahme erheblich (Präsenzlehre: M = 2,44–1,81; digitale Lehre: M = 2,58–1,51; alle Paarvergleiche *p* < 0,001; Abb. 2).

Es konnten weiterhin zwei signifikante Interaktionseffekte zwischen Lehrform und Lernzuwachs gefunden werden: Die Lernzuwächse bei (c) „psychotherapeutischen Verfahren“ (GLM: F[1, 328] = 11,52, *p* < 0,001, partielles η^2^ = 0,03) und (f) „Schizophrenie“ (GLM: F[1, 326] = 9,55, *p* = 0,002, partielles η^2^ = 0,03) waren bei der digitalen Lehre signifikant höher ausgeprägt als bei der Präsenzlehre (Abb. 2).

### Vergleichende Evaluation: inhaltliche Bewertung

Die digitale Lehre korrelierte mit besseren inhaltlichen Bewertungen in 5 Bereichen (r = −0,13 bis −0,41, *p* < 0,05 bis < 0,01; s. Tab. 3 für eine Übersicht). Die Studierenden konnten zudem eine Schulnote für die jeweilige Lehrform in ihrem Semester vergeben. Hierbei schnitten sowohl die Präsenzlehre mit M = 2,05 (SD = 0,81) als auch die digitale Lehre mit M = 1,86 (SD = 0,71) tendenziell mit der Note „gut“ ab.

**Table 3:** 

Variable	1	2	3	4	5	6	7	8	9	M ± SD/Freq
1. Digital										j: 175, n : 175
2. Arbeitsstunden	0,396^**^									32,11 ± 12,97
3. Interdisziplinäre Umsetzung	−0,102	0,111								2,09 ± 1,06
4. Selbstständiges Aufarbeiten von Lernziele	−0,4n14^**^	−0,105	0,524^**^							2,02 ± 1,12
5. Berufliche Zukunft: Lernzuwachs	−0,074	0,044	0,477^**^	0,402^**^						1,91 ± 0,98
6. Struktur des Moduls: Zufriedenheit	−0,157^**^	0,022	0,564^**^	0,388^**^	0,480^**^					2,25 ± 1,33
7. Praktische Umsetzung der Lehre	−0,157^**^	0,083	0,420^**^	0,379^**^	0,411^**^	0,420^**^				1,77 ± 1,04
8. Wunsch nach Fortführung in der Form	−0,134^*^	−0,064	0,474^**^	0,344^**^	0,415^**^	0,588^**^	0,571^**^			2,23 ± 1,37
9. Vorlesungen: Lernerfolg	−0,315^**^	−0,104	0,235^**^	0,215^**^	0,299^**^	0,264^**^	0,330^**^	0,425^**^		2,02 ± 1,24
10. Seminare: Lernerfolg	−0,093	−0,005	0,164^**^	0,186^**^	0,209^**^	0,156^**^	0,201^**^	0,234^**^	0,331^**^	2,44 ± 1,37

*M* Mittelwert, *SD* Standardabweichung, *Freq* Frequenz

Digital: 0 = Präsenzlehre, 1 = digitale Lehre; Arbeitsstunden für das Modul in Zeitstunden; Items 3 bis 10: Bewertung der Lehre (1 = „trifft voll zu“ bis 6 = „trifft überhaupt nicht zu“); für Details zu den Items s. Abschnitt „Fragebogen zur Lehrevaluation“; *n* = 350; df = 224–348

**Figure 3:**
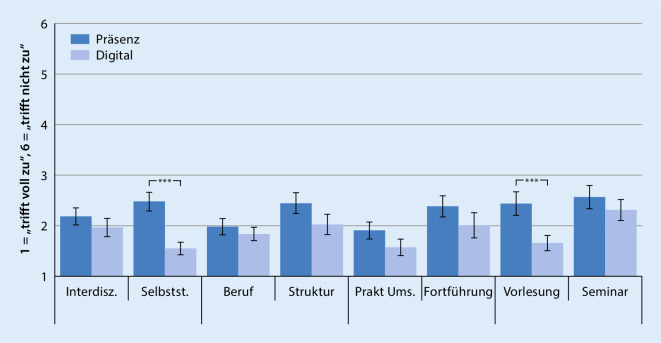

* *p* < 0,05; ** *p* < 0,01;

Die Studierenden gaben an, signifikant mehr wöchentliche Arbeitsstunden in die digitale Lehre (M = 37,18, SD = 10,68) im Vergleich zur Präsenzlehre investiert zu haben (M = 26,93, SD = 13,09, t[275] = 7,14, *p* < 0,001). Bezogen auf die inhaltlichen Bewertungen konnten signifikante Unterschiede zugunsten der digitalen Lehre in 2 von 8 Bereichen gefunden werden (Abb. 3): „Selbstständiges Aufarbeiten von Lernzielen“ (t[315] = 8,08, *p* < 0,001), „Beitrag der Vorlesung zum Lernerfolg“ (t[290] = 5,66, *p* < 0,001). In allen übrigen Bereichen ergaben sich zwar numerische Unterschiede zugunsten der digitalen Lehre, diese verfehlten jedoch die Signifikanz (Abb. 3).

## Diskussion

In der vorliegenden Studie wurde untersucht, ob eine kurzfristige Digitalisierung der psychiatrischen Lehre im Rahmen der Corona-Pandemie zu ähnlichen Lernerfolgen bei den Studierenden führte wie die etablierte Präsenzlehre. Zudem schätzten Studierende die digitale Lehre in Bezug auf Aufwand und Relevanz für Staatsexamen und die spätere ärztliche Praxis ein. Literatur zu einer solch kurzfristig notwendigen Digitalisierung der Lehre ist bislang nicht existent, die vorliegende Studie liefert hierzu erste empirische Daten.

Durch die digitale Lehre wurde ein gleichwertiger subjektiver *Lernzuwachs* im Vergleich zur Präsenzlehre erreicht, teils darüber hinaus (Teilbereiche „Psychotherapie“, „Schizophrenie“). Eine mögliche Erklärung hierfür ist der erhöhte Arbeitsaufwand (ca. 10 Zeitstunden) in der digitalen Lehre. Nachgewiesenermaßen sind aber auch Arbeitstechniken und Lernstrategien [15], spezifische Vorkenntnisse [12] sowie die Lernmotivation [3] wichtige Einflussgrößen auf den Lernerfolg. Daher scheint die Annahme gerechtfertigt, dass neben dem höheren Arbeitsaufwand auch die neu etablierten Lehrkonzepte (Podcasts, Seminare als „inverted classrooms“) zum Lernerfolg beigetragen haben. Hierzu passend wurde der Beitrag der Vorlesungspodcasts zum Lernerfolg durch die Studierenden höher eingeschätzt, als es in den vergangenen Semestern für die Präsenzvorlesungen der Fall war.

Bemerkenswerterweise führte die deutlich höhere wöchentliche zeitliche Belastung nicht zu einer schlechteren* inhaltlichen Bewertung* seitens der Studierenden; beide Lehrformen wurden tendenziell als „gut“ bewertet. Auch die Abrufzahlen der Podcasts (Vorlesungspodcasts: 57 %, Seminarpodcasts: 75 %) können vor dem Hintergrund, dass kein Nachweis des Abrufs zur Scheinvergabe notwendig war, auch im Vergleich zu anderen Studien, als hoch angesehen werden [7]. Insgesamt kann auf eine hohe Akzeptanz der Studierenden (trotz erhöhten Aufwandes) für die digitale Lehre geschlossen werden, was auch in anderen Studien gezeigt werden konnte [11]. Auch die weitere vergleichende Bewertung fiel äquivalent bzw. zugunsten der digitalen Lehre aus: Insbesondere das „selbstständige Aufarbeiten von Lernzielen“ und die Vorlesung wurden in der digitalen Lehre von den Studierenden besser bewertet.

Die von den Studierenden in der Modulklausur erreichte Durchschnittsnote fiel mit 1,8 bei vergleichbarer Schwierigkeit (0,8439) und erhöhter Trennschärfe (0,4281) etwas besser aus als in den vorangegangenen Semestern (Durchschnittsnoten: WS 18/19: 2,8; SoSe 19: 2,1; WS 19/20: 2,0). Allerdings war bereits über die letzten Semester eine Tendenz zur besseren Durchschnittsnote zu erkennen, sodass unklar bleibt, ob die digitale Lehrform einen direkten Einfluss auf die Klausurergebnisse hatte.

Skepsis zeigten die Studierenden insbesondere bez. der Frage, ob die digitale Lehre eine bessere Vorbereitung auf den ärztlichen Beruf bieten würde: Dies wurde tendenziell verneint. Hierbei ist jedoch zu beachten, dass zum Zeitpunkt der Evaluation für alle Beteiligten noch nicht absehbar war, ob und in welcher Form der Unterricht am Krankenbett (UAK) im Sommersemester 2020 durchgeführt werden konnte. Somit ergab sich für die Studierenden die Perspektive, im digitalen Semester nicht in direkten Kontakt mit „echten“ psychiatrischen Patienten zu kommen. Dies könnte zur Einschätzung beigetragen haben, schlechter auf den ärztlichen Berufsalltag vorbereitet zu sein. Trotz der insgesamt positiven Einschätzung, gerade im Hinblick auf die Examensvorbereitung, scheinen die Studierenden die alleinige digitale Lehre als nur unzureichende Vorbereitung auf die spätere Tätigkeit zu sehen. Die klassische Präsenzlehre sollte daher auch in zukünftigen Kurrikula fest verankert und durch digitale Lehrangebote sinnvoll ergänzt werden.

### Limitationen

(1) Für die hier vorliegende vergleichende Evaluation wurde der Lernzuwachs von den Studierenden jeweils nur einmal eingeschätzt (Post-hoc-Erhebung), dementsprechend ist die rückblickende Selbsteinschätzung *vor *Modulbeginn mit hoher Wahrscheinlichkeit weniger präzise (Abb. 2; erhöhte Konfidenzintervalle bei rückblickender Einschätzung). In Zukunft wäre eine längsschnittliche Erhebung vor und nach Durchführung des Moduls wünschenswert, um den Messfehler zu minimieren. (2) Eine detaillierte Stichprobenbeschreibung ist nicht möglich: Zwar kann durch den hohen Stichprobenumfang von einer ausreichenden Repräsentativität bez. der studentischen Population ausgegangen werden, jedoch sind auf Basis der standardisierten Lehrevaluation mangels zulässiger Erfassung keine Aussagen zu demographischen Subgruppen möglich. (3) Auffällig ist die erheblich geringere Teilnahmequote der Studierenden an der freiwilligen Lehrevaluation der Präsenzlehre (ca. 41 %) im Vergleich zur digitalen Lehre (ca. 98 %) – ein Bias zugunsten der digitalen Lehre kann somit nicht ausgeschlossen werden. Dies ist möglicherweise durch die unterschiedliche Durchführung der Lehrevaluation bedingt: In Semestern mit Präsenzlehre erhielten die Studierenden im Anschluss an die Klausur einen Link zugeschickt, über welchen die Lehrevaluation auszufüllen war. Im Sommersemester 2020 wurden die Evaluationsbögen hingegen ausgedruckt und mit den Klausurbögen ausgeteilt. Die Hürde zum Ausfüllen der Lehrevaluation war somit für die Studierenden im Sommersemester kleiner.

## Schlussfolgerung und Fazit für die Praxis

Die pandemiebedingte Umstellung der Präsenzlehre hin zur digitalen Lehre scheint ohne Qualitätsverlust möglich. Durch den Einsatz neuer Lehrformate (Vorlesungspodcasts, Seminare in Form des „inverted-classrooms“) können – bei erhöhtem Arbeitsaufwand seitens der Studierenden – gleichwertige subjektive Lernerfolge und hohe Akzeptanz erzielt werden. Mit Blick auf die spätere praktische ärztliche Tätigkeit sollten in zukünftigen Kurrikula neben der klassischen Präsenzlehre ergänzend digitale Lehrangebote verankert werden.

Durch positive Erfahrungen mit der digitalisierten Lehre können Vorbehalte auf Seiten der Lernenden, insbesondere jedoch auf Seiten der Studierenden abgebaut werden. Dies und mögliche Weiterbildungen im Umgang mit neuen Lehrmethoden sollten die Voraussetzung für eine langfristige Etablierung der digitalen Lerninhalte im Medizinstudium darstellen.

## Footnotes

1 Da der Unterricht am Krankenbett unter bestimmten Auflagen ab dem 22.06.2020 doch in Präsenzform durchgeführt werden konnte, wurde diese Unterrichtsform für diesen Vergleich nicht berücksichtigt.

2 Beispiel: „Ich kann die verschiedenen Formen der Angststörungen unterscheiden.“ Die Einschätzung erfolgte für die beiden Items: „*aktuelle* Selbsteinschätzung“ vs. „*rückblickende* Selbsteinschätzung zu Modulbeginn“.

3 Beispiel: „Die *Seminare der Psychiatrie* in diesem Modul haben wesentlich zu meinem Lernerfolg beigetragen.“.

4 Beispiel: „Im Vergleich zur Präsenzlehre bin ich durch die digitale Lehre besser auf den ärztlichen Beruf vorbereitet.“.
